# Editorial: PLP-Dependent Enzymes: Extraordinary Versatile Catalysts and Ideal Biotechnological Tools for the Production of Unnatural Amino Acids and Related Compounds

**DOI:** 10.3389/fbioe.2020.00052

**Published:** 2020-02-11

**Authors:** Martino L. Di Salvo, Kateryna Fesko, Robert S. Phillips, Roberto Contestabile

**Affiliations:** ^1^Laboratory Affiliated to Istituto Pasteur Italia-Fondazione Cenci Bolognetti, Dipartimento di Scienze Biochimiche, Sapienza Università di Roma, Rome, Italy; ^2^Institute of Organic Chemistry, Graz University of Technology, Graz, Austria; ^3^Department of Chemistry and Department of Biochemistry and Molecular Biology, University of Georgia, Athens, GA, United States

**Keywords:** PLP-dependent enzymes, enzyme catalysis, unnatural (non-canonical) amino acids, green chemistry, biotechnology

The main focus of this *Frontiers Research Topic* is on the most recent structural, functional and computational studies on pyridoxal-5′-phosphate (PLP)-dependent enzymes and their potential use for the production of unnatural amino acids.

The biocatalytic asymmetric synthesis of unnatural amino acids and related compounds has been the topic of great attention in recent years (Bornscheuer, 2018). In living organisms, enzymes that use PLP—the active form of vitamin B_6_–as cofactor are able to catalyze a wide variety of biochemical reactions involving amino acids substrates and their analogs. These enzymes show an unparalleled catalytic versatility, essentially due to the chemistry of their cofactor, and catalyze a number of diverse chemicals reactions, such as decarboxylation, transamination, racemization, β- and γ-elimination and substitution, carbon-carbon bond cleavage and formation, as well as oxidation (Eliot and Kirsch, 2004; Paiardini et al., 2014). For these reasons, PLP-enzymes have drawn interest as powerful tool in biotechnology, for the *green chemistry* production of a whole range of specific amino acids and their derivatives. Furthermore, engineering of metabolic pathways mediated by PLP-dependent enzymes is a promising approach to provide cells with unnatural amino acids, produced intracellularly from simple carbon sources or precursors (Ma et al., 2014).

Amino acids and amines are among the most important molecules in nature and chemistry. They play central roles both as intermediates in metabolism and as building blocks of proteins and pharmaceuticals. By incorporating an alternative amino acid into proteins, the available chemical options to achieve additional functionalities is substantially enlarged (Budisa, 2013). For example, halogenated tyrosine and azatryptophan analogs can be used to alter catalytic activity profile or intrinsic protein fluorescence (Lepthien et al., 2008; Wilkins et al., 2010); hydroxyl amino acids can be site-specifically installed into proteins to define specific sites for chemical hydrolysis (Kobayashi et al., 2009); in addition, unnatural amino acids can be used to derivatize proteins with PEG molecules, sugars, oligonucleotides, fluorophores, peptides, and other unique synthetic moieties (Budisa, 2005; Liu et al., 2011); special unnatural amino acids, e.g., fluorinated analogs or sterically hindered α-amino acids, have highly attractive unique features in drug discovery and development, synthesis of active pharmaceutical ingredients, as well as in disease diagnostics as potential biomarkers (Budisa et al., 2010).

So far, the described protocols for the asymmetric synthesis of unnatural amino acids using PLP-enzymes include application of threonine aldolases (Blesl et al., 2018) for the synthesis of L- and D-β-hydroxy amino acids, kynureninase, tryptophanase, tyrosine phenol-lyase, and tryptophan synthase for the synthesis of aromatic amino acids, O-acetyl(homo)serine sulfhydrylase for the synthesis of (homo)cysteine derivatives, and a wealth of protocols describing stereoselective synthesis of amino acids and amines using PLP-dependent transaminases (Di Salvo et al., 2012; Rocha et al., 2019). However, many PLP-dependent enzymes are still poorly investigated as potential catalysts. PLP-dependent enzymes from extremophile organisms are of particular interest in this context (Angelaccio, 2013).

This *Frontiers Research Topic* puts the focus on new knowledge on PLP-dependent activities, structure-function relationships, catalytic mechanisms, reaction, and substrate specificity of this class of enzymes:

- Two new thermophilic branched chain amino acid aminotransferases have been identified by Isupov et al., within the genomes of hyper-thermophilic archaea *Geoglobus acetivorans* and *Archeaglobus fulgidus*. The enzymes have been characterized both biochemically and structurally and show optimum activity temperatures between 80 and 85°C. Interestingly, they retain catalytic activity after exposure to up to 50% of organic solvents, ethanol, methanol DMSO and acetonitrile, and are able to accept substrate analogs such as methylbenzylamine in a stereospecific manner. Both enzymes have been crystallized in the apo-form and in complex with substrates and inhibitors, in order to better understand turnover mechanism and substrate specificity;
- A putative PLP-dependent aminotransferase from *Aspergillus fumigatus*–an opportunistic fungus responsible for lethal invasive infections–whose gene regulation by L-tryptophan is involved in the fungus-host interaction, has been biochemically characterized by Dindo et al. The enzyme was proved to be an aromatic amino acid aminotransferase, also accepting L-kynurenine and α-aminoadipate as substrates. The study, targeting the therapeutic value of fungal metabolic adaptation, aims toward the design of specific modulators as potential novel agents to interfere with the fungus-host interaction;
- An overview on PLP-dependent enzymes from the Gram-positive soil bacterium *Bacillus subtilis* (a model system in basic research and a production host in bioindustry) has been performed by Richts et al., which could facilitate bacteria engineering for biotechnological applications. In this search, a surprisingly large group of PLP-dependent proteins, about 29%, have been found to be poorly characterized, supporting the importance of better investigation of these proteins of unknown function to fully understand the “PLP-ome” of this model organism;
- A carbon acidity study of substrates at the enzyme active site was performed by Toney, allowing calculation of pK_a_s for 11 enzymes, among them several PLP-dependent enzymes. Active site-bound substrate C-H pK_a_ values ranged from 5.6 to 16. Compared to pKa values in water, enzymes lower substrate C-H pK_a_s by up to 23 units, corresponding to as much as 31 kcal/mol of carbanion stabilization energy. The Marcus intrinsic barriers ($\Delta G_{0}^{‡}$) for non-enzymatic vs. enzymatic reactions show significant reductions for cofactor-independent enzymes, while PLP-dependent enzymes appear to increase $\Delta G_{0}^{‡}$ to a small extent, as a consequence of carbanion resonance stabilization. The large increase in carbon acidity found here is central to the large rate enhancement observed in enzymes that catalyze carbon deprotonation;

Also, the intrinsic catalytic promiscuity of the PLP cofactor, in the scaffold of existing PLP-dependent enzyme structures, has been exploited to develop new catalytic functions. This is a promising approach for the asymmetric synthesis of chiral compounds. Especially, ω-transaminases have shown great promise as versatile industrial biocatalysts with high selectivity, regioselectivity, and broad substrate scope. The toolbox of transaminases for the stereoselective synthesis of amines and amino acids has been enormously expanded in the last years by a number of natural and genetically modified enzymes.

- In this frame, Galman et al. have identified putrescine transaminase from *Pseudomonas putida* as a biocatalyst for the direct reductive amination of prochiral ketones to access chiral amines. This enzyme was biochemically and structurally characterized to reveal its high relative activity at 60°C, and excellent stability. Putrescine transaminase was shown to accept cadaverine or isopropylamine as equilibrium shifting amine donors, and a comprehensive range of ketones and aldehydes for the production of a variety of benzylamine derivatives with excellent product conversion and extremely broad substrate tolerance. This method also eliminates the need for an expensive cofactor recycling system, making this approach greener, and more economically feasible.

Taken together, these studies highlight once more the importance of PLP-dependent enzymes as ideal biotechnological tools.

## Author Contributions

All authors listed have made a substantial, direct and intellectual contribution to the work, and approved it for publication.

### Conflict of Interest

The authors declare that the research was conducted in the absence of any commercial or financial relationships that could be construed as a potential conflict of interest.
